# 6‐Gingerol, an active pungent component of ginger, inhibits L‐type Ca^2+^ current, contractility, and Ca^2+^ transients in isolated rat ventricular myocytes

**DOI:** 10.1002/fsn3.968

**Published:** 2019-02-19

**Authors:** Xue Han, Yuanyuan Zhang, Yingran Liang, Jianping Zhang, Mengying Li, Zhifeng Zhao, Xuan Zhang, Yurun Xue, Ying Zhang, Jingkai Xiao, Li Chu

**Affiliations:** ^1^ School of Pharmacy Hebei University of Chinese Medicine Shijiazhuang China; ^2^ School of Basic Medicine Hebei University of Chinese Medicine Shijiazhuang China; ^3^ Hebei Key Laboratory of Integrative Medicine on Liver‐Kidney Patterns Shijiazhuang China

**Keywords:** 6‐Gingerol, Ca^2+ ^transients, cardiomyocytes, contractility, L‐type Ca^2+^ current

## Abstract

Ginger has been widely used as a flavor, food, and traditional medicine for centuries. 6‐Gingerol (6‐Gin) is the active components of ginger and offers some beneficial effects on cardiovascular diseases. Here, the effects of 6‐Gin on L‐type Ca^2+^ current (I_Ca‐L_), contractility, and the Ca^2+ ^transients of rat cardiomyocytes, were investigated via patch‐clamp technique and the Ion Optix system. The 6‐Gin decreased the I_Ca‐L _of normal and ischemic ventricular myocytes by 58.17 ± 1.05% and 55.22 ± 1.34%, respectively. 6‐Gin decreased I_Ca‐L _in a concentration‐dependent manner with a half‐maximal inhibitory concentration (IC_50_) of 31.25 μmol/L. At 300 μmol/L, 6‐Gin reduced the cell shortening by 48.87 ± 5.44% and the transients by 42.5 ± 9.79%. The results indicate that the molecular mechanisms underlying the cardio‐protective effects of 6‐Gin may because of a decreasing of intracellular Ca^2+^ via the inhibition of I_Ca‐L_ and contractility in rat cardiomyocytes.

## 1 INTRODUCTION

Ginger belongs to the Zingiberaceae family with a long history of use as a flavor and a food. Ginger has also been used medicinally for indigestion, vomiting, arthritis, fever, pains, cramps, etc (Ali, Blunden, Tanira, & Nemmar, 2008). It is used as a traditional medicine in South Asia for cardiopathy and hypertension (Ghareib et al., 2015). It has active ingredients that mediate its effects, and ginger extracts are used extensively in beverages, liquors, pickles, and so on (Wohlmuth, Leach, Smith, & Myers, 2005).

**Figure 1:**
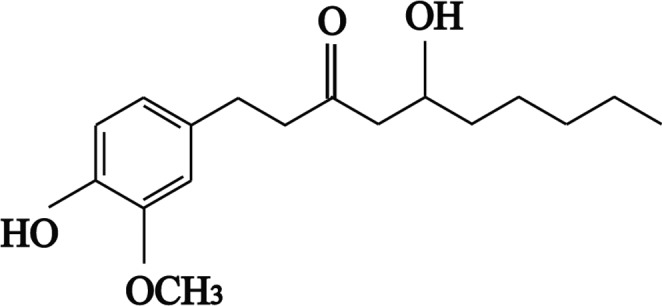
Chemical structure of 6‐Gin

Ginger contains more than 80 types of vanilloids including gingerols and shogaols (Jolad, Lantz, Chen, Bates, & Timmermann, 2005). Gingerols are one of the most common active components (Koo, Ammit, Tran, Duke, & Roufogalis, 2001). Gingerols are thermally sensitive and dehydrated to 6‐, 8‐, and 10‐shogaol at high temperature (Ezzat, Ezzat, Okba, Menze, & Abdel‐Naim, 2018; Kou et al., 2018). The major bioactive constituent of ginger is 6‐gingerol (6‐Gin) (Shukla & Singh, 2007), 8‐gingerol, and 10‐gingerol account for only a fraction. 6‐Gin is the most abundant and pungent gingerol in ginger, and its structural formula was shown in Figure 1. It has diverse and interesting pharmacological effects including antipyretic, anti‐inflammatory, anti‐angiogenic, anti‐cancer, cardio‐tonic, and anti‐aging effects. It inhibits spontaneous motor activity and prostaglandin biosynthesis (Ajayi, Adedara, & Farombi, 2018; Dugasani et al., 2010; Kim et al., 2005; Kiuchi, Iwakami, Shibuya, Hanaoka, & Sankawa, 1992; Lee et al., 2018; Lee, Seo, Kang, & Kim, 2008; Lv et al., 2018; Suekawa et al., 1984; Tahir et al., 2015).

External Ca^2+^ enters the cell by passing through calcium channels. Ca^2+ ^can also be released from internal Ca^2+^ stores including the endoplasmic reticulum (ER) or sarcoplasmic reticulum (SR) (Berridge, 1993; Clapham, 1995). This area houses protein synthesis and transport to membranous networks. The Ca^2+^ mainly enters through L‐type Ca^2+^channels (LTCCs), which are essential to cardiac excitability and excitation–contraction coupling (Ferrier & Howlett, 2001).

In cardiacmyocyte, Ca^2+^ activates the ryanodine receptor (RYR2) to create the “spark” triggering contraction. Contractility is related to intracellular Ca^2+ ^([Ca^2+^]_i_) and the sensitivity of myofilaments to Ca^2+^. There is ample evidence that excess Ca^2+ ^can produce pathological changes in cardiac tissue (Grinwald, 1982; Harding & Poole‐Wilson, 1980; Sharma, Saffitz, Lee, Sobel, & Corr, 1983; Shen & Jennings, 1972) such as increased contractility, hypertrophy (Frey & Olson, 2003), and apoptosis (Chen et al., 2005). In addition, the increased contractility of cardiomyocyte can lead to ischemic myocardial diseases (Gao et al., 2014).

L‐type Ca^2+^channels are related to Ca^2+^ influx (Liu et al., 2016). Therefore, LTCCs blockers generally protect against myocardial ischemic injury via the inhibition of calcium channels. Previous studies have emphasized the inhibitory effect of verapamil (VER) on myocardial contraction and the protective effect on excess calcium overload (Song et al., 2017). Hence, drugs that can weaken I_Ca‐L_ are promising for myocardial protection (Song et al., 2016).

Recent reports have detailed the cardio‐protective effect of 6‐Gin against ischemia–reperfusion injury in rats (Lv et al., 2018); however, the precise mechanism underlying the cellular Ca^2+^ homeostasis remains poorly understood. The pathogenesis of ischemic disease is related to Ca^2+^ signaling and cardiac function; thus, it is important to explain the direct action of 6‐Gin on Ca^2+^ homeostasis and contractility in cardiomyocytes as well as the potential character of 6‐Gin on treatment of Ca^2+^‐related cardiac disease. This work systematically characterized the regulatory effects of 6‐Gin on L‐type Ca^2+^ current (I_Ca‐L_), contractility, and Ca^2+^ transients in isolated rat ventricular myocytes via the patch‐clamp technique and the Ion Optix system. It further explored the possible cellular mechanism of 6‐Gin for the management of ischemic cardiac diseases.

## 2 MATERIAL AND METHOD

### 2.1 Chemicals

6‐Gin was purchased from Yuanye Biotechnology Co., Ltd (China). Type Ⅱcollagenase was bought from Worthington Biochemical Corporation (USA). VER was from Hefeng Pharmaceutical Co., Ltd. (China). Other chemicals and reagents were acquired from Sigma (USA) and were of analytical grade.

### 2.2 Animals

Male Sprague‐Dawley rats (180–220 g) were from the National Experimental Animal Center of Hebei, National Science Council. They were housed in cages at a constant temperature of 25 ± 1°C and supplied with food and water (approval number: 1803064; approval date: March 7, 2018).

### 2.3 Isolation of ventricular myocytes

Rat ventricular myocytes were isolated via Mitra and Morad (Mitra & Morad, 1985). Briefly, rats were injected with heparin (1,500 IU/kg, i.p.) and anesthetized with sodium urethane (40 mg/kg, i.p.). The hearts were then quickly excised and perfused at 6 ml/min with Ca^2+^‐free Tyrode solution for 4 min and Ca^2+^‐free Tyrode's solution containing CaCl_2_ (34 μmol/L) and collagenase (500 mg/L) for 15–20 min via Langendorff equipment. The hearts were then washed with Tyrode's solution after the digestion. The freshly dissociated cells were stored in Kreb's buffer solution.

Rats were injected with vasopressin via tail vein (1.5 IU/kg, i.v.) to induce cardiac ischemia (Li et al., 2014). After 10 min of ischemia, the heart was removed as above to isolate normal rat ventricular myocytes.

### 2.4 Measurement of I_Ca‐L_

The Ca^2+^‐current was recorded via the whole cell patch‐clamp10.0 software using an Axon patch 200B amplifier (Axon Instrument, USA). The patch electrodes were pulled with a pipette puller (Sutter Instruments, USA). By recording the I_Ca‐L_, the external solution contained (in mmol/L) TEACl 140, MgCl_2_ 2, CaCl_2_ 1.8, glucose 10, and HEPES 10, and the pH was adjusted to 7.4 with CsOH. The intracellular pipette solution contained (in mmol/L) CsCl 120, tetraethylammonium chloride (TEACL) 20, HEPES 10, Mg‐ATP 5, and EGTA 10, and the pH was adjusted to 7.2 with CsOH. Drugs were dissolved in Tyrode's solution.

### 2.5 Measurement of contractility

The contractions of ventricular myocytes were recorded with a video‐based edge‐detection system (Ion Optix, USA). Cells were placed on the stage of inverted microscope, and contractility was induced at a frequency of 0.5 Hz. Clear myocytes were selected to measure contractions.

### 2.6 Measurement of Ca^2+^ transients

Fura‐2/AM (1 mmol/L) was fitted with a 340 or 380 nm optical filter and used to study ventricular myocyte [Ca^2+^]_i_ dynamics and associated myocyte contractile function. Ventricular myocyte was loaded with the fluorescent dye in the dark and measured with a fluorescence system (Ion Optix). The contractility of the myocytes was stimulated with a 0.5 Hz field.

### 2.7 Data analysis

The results were presented as mean ± *SEM*. Comparisons were analyzed via one‐way analysis of variance (ANOVA) followed by the Student's *t* test using Origin Pro version 9.1 software. *p < *0.05 was considered to be statistically significant.

## 3 RESULTS

### 3.1 Confirmation of I_Ca‐L_

**Figure 2:**
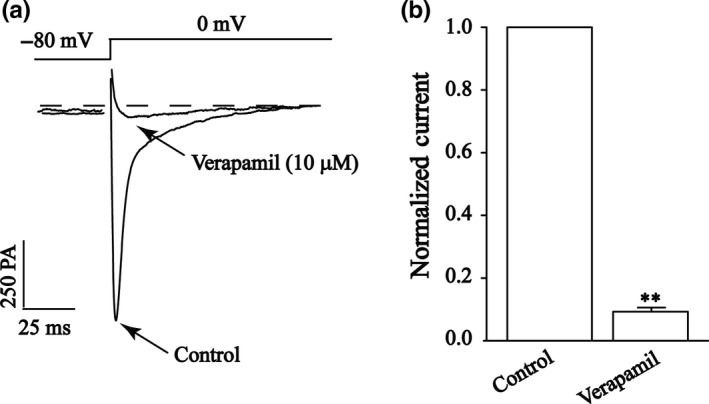
Confirmation of I_Ca‐L_ in cardiomyocytes. (a) Exemplary traces and (b) pooled data showed the representative I_Ca‐L_ recordings with application of VER (10 μmol/L). Data are expressed as mean ± *SEM* (*n* = 5 cells). **p < 0.01 versus control

Verapamil (10 μmol/L) is a specific I_Ca‐L_ blocker and nearly completely stopped current flow (*p < *0.01) (Figure 2), indicating that the L‐type channels are functional in cardiomyocytes.

### 3.2 Effects of 6‐Gin on I_Ca‐L _of normal and ischemic ventricular myocytes

**Figure 3:**
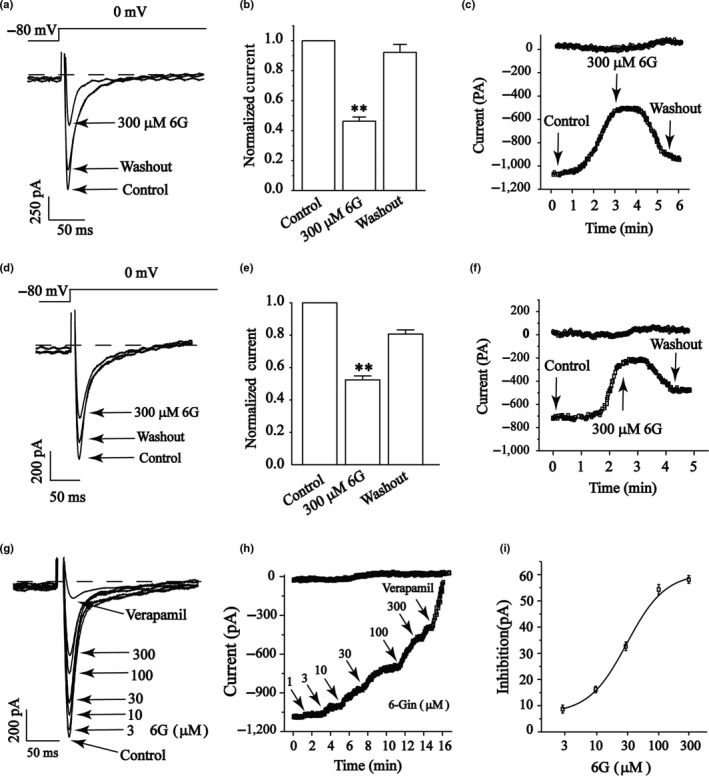
Reversible effects of 6‐Gin on I_Ca‐L _in normal ventricular myocytes and ischemic ventricular myocytes. Exemplary traces (a, d), pooled data (b, e), and time course (c, f) of I_Ca‐L _were measured under the treatment of 6‐Gin (300 μmol/L) and during washout. (g) Exemplary traces and (h) time course of I_Ca‐L _in exposure to 3, 10, 30, 100, 300 μmol/L 6‐Gin or 10 μmol/L VER. (i) Concentration‐response curves of 6‐Gin. Data are expressed as mean ± *SEM* (*n* = 6–8 cells). **p < 0.01, versus control

Figure 3 shows that 6‐Gin (300 μmol/L) significantly reduced the I_Ca‐L _of normal (Figure 3a–c) and ischemic ventricular myocytes (Figure 3d–f) by 58.17 ± 1.04% and 55.22 ± 1.34%, respectively (*p < *0.01). Nevertheless, the I_Ca‐L_ partially recovered after washing with an external solution, suggesting reversible effects of 6‐Gin on the I_Ca‐L _of normal and ischemic ventricular myocytes. The time course of I_Ca‐L_ was progressively decreased by increasing doses of 6‐Gin (3, 10, 30, 100, and 300 μmol/L) or VER (Figure 3g). The time dependency of the 6‐Gin on I_Ca‐L _is shown in Figure 3h. The half‐maximal inhibitory concentration (IC_50_) of 6‐Gin was 31.25 μmol/L. The inhibition rates of 6‐Gin at 3, 10, 30, 100, and 300 μmol/L were 8.71 ± 0.60%, 16.2 ± 0.8%, 32.67 ± 0.76%, 54.33 ± 1.89%, and 58.17 ± 1.04%, respectively (Figure 3i).

### 3.3 Effects of 6‐Gin on I‐V relationship of I_Ca‐L_

**Figure 4:**
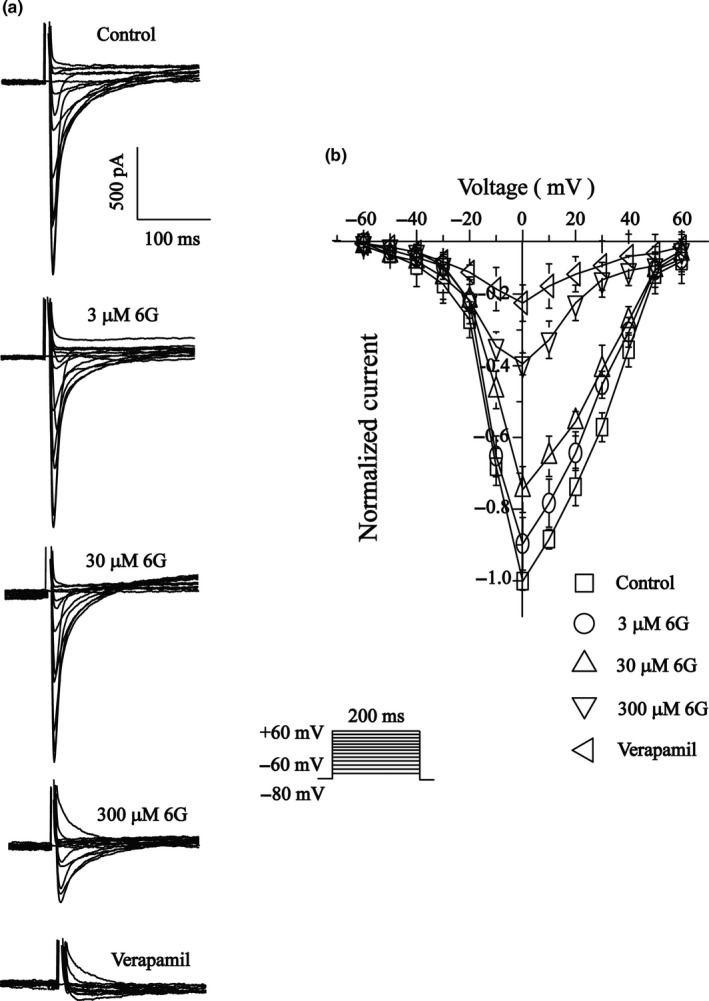
Effects of 6‐Gin on I‐V relationship of I_Ca‐L_. Representative I_Ca‐L _(a) and pooled data (b) are shown under the treatment of control (□), 6‐Gin at 3 μmol/L (○), 30 μmol/L (△), 30 μmol/L TG (▽) or VER at 10 μmol/L (◇). Data are expressed as means ± *SEM* (*n* = 8 cells)

**Figure 5:**
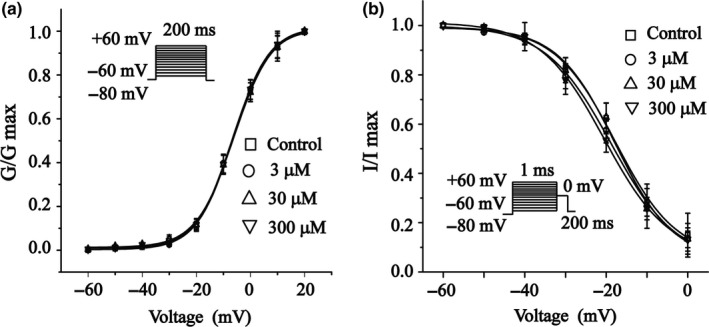
Effects of 6‐Gin on steady‐state activation and inactivation of I_Ca‐L_. Steady‐state activation curves (a) and inactivation curves (b) of I_Ca‐L _are shown under the treatment of control (□), 6‐Gin at 3 μmol/L (○), 30 μmol/L (△), 300 μmol/L TG (▽). Data are expressed as means ± *SEM* (*n* = 8 cells)

Figure 4a shows the current–voltage relationship curves for different concentrations of 6‐Gin (3, 30, and 300 μmol/L) and VER (10 μmol/L). Figure 5b shows the current generated from −60 to 60 mV. Nevertheless, the I‐V relationship and reversal potential of I_Ca‐L _did not change significantly.

### 3.4 Effects of 6‐Gin on steady‐state activation and inactivation of I_Ca‐L_

Figure 5 shows the effects of 6‐Gin concentrations (3, 30 and 300 μmol/L) on steady‐state activation and inactivation of I_Ca‐L_. The V_1/2_ value for activation of 3, 30, and 300 μmol/L 6‐Gin was −6.54 ± 0.28 mV/6.70 ± 0.24, −6.66 ± 0.28 mV/6.78 ± 0.25, −6.14 ± 0.29 mV/6.96 ± 0.26, and −6.44 ± 0.28 mV/6.71 ± 0.25, respectively. The V_1/2_ value for inactivation of 0, 3, 30, and 300 μmol/L 6‐Gin was −17.78 ± 1.17 mV/7.44 ± 0.98, −18.80 ± 1.22 mV/8.01 ± 1.06, and −17.81 ± 1.06 mV/7.08 ± 0.09, −2,046 ± 1.11 mV/7.89 ± 0.99, respectively.

### 3.5 Effects of 6‐Gin on Ca^2+ ^contractility

**Figure 6:**
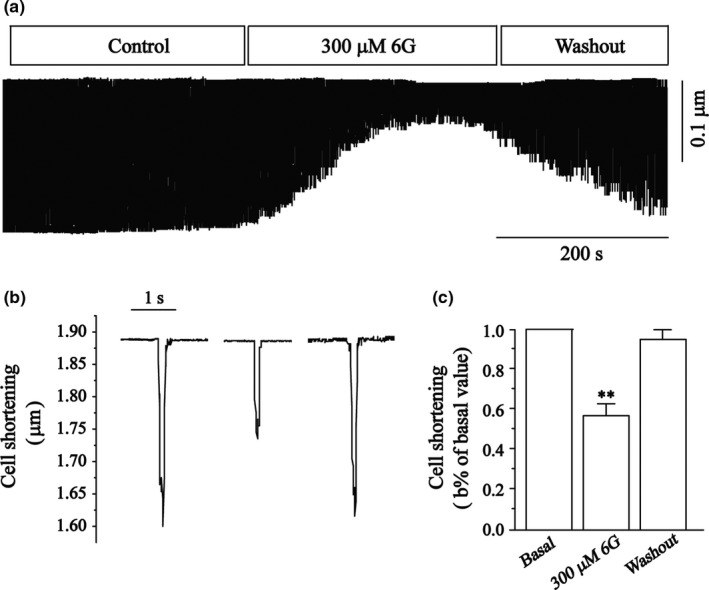
Effects of 6‐Gin on contractility in ventricular myocytes. (a) Recordings of contractility on time course in the absence and presence of 6‐Gin (300 μmol/L). (b) Exemplary traces recordings of contractility under control conditions and 6‐Gin (300 μmol/L). (c) Summary data of contractility before and after treatment of 300 μmol/L 6‐Gin. Data are expressed as means ± *SEM* (*n* = 6–8 cells). **p < 0.01, versus control

Changes in the 6‐Gin on myocyte shortening are shown in Figure 6. 6‐Gin (300 μmol/L) significantly inhibited myocyte shortening by 48.87 ± 5.44%. The contractility recovered partially after washing out.

### 3.6 Effects of 6‐Gin on transients

**Figure 7:**
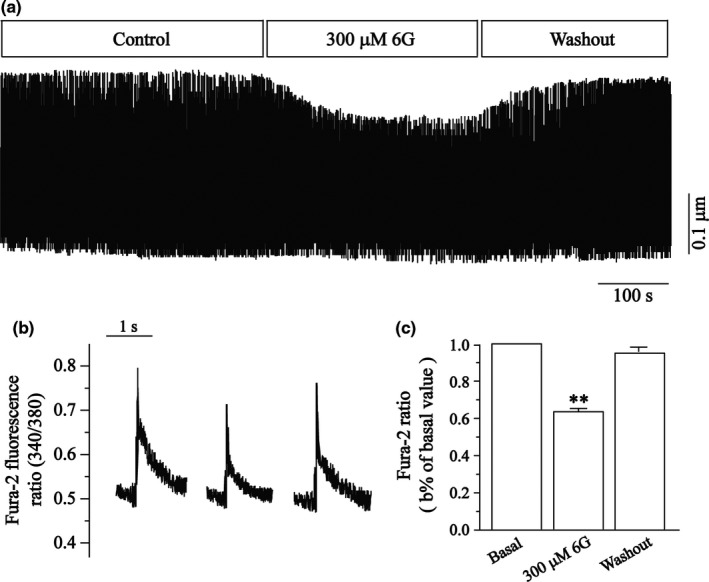
Effects of 6‐Gin on Ca^2+^ transients. (a) Recordings of time course of Ca^2+^ transient in the absence and presence of 6‐Gin (300 μmol/L). (b) Recordings of exemplary traces of Ca^2+^ transients under control conditions and 300 μmol/L 6‐Gin. (c) Summary data of Ca^2+^ transients before and after application of 300 μmol/L 6‐Gin. Data are expressed as means ± *SEM* (*n* = 6 cells). **p < 0.01, versus control

The changes of the 6‐Gin on Ca^2+^ transients are shown in Figure 7. The 6‐Gin (300 μmol/L) significantly inhibited the Ca^2+^ transients by 42.5 ± 9.79%. The transients partially recovered partially after washing.

### 3.7 Effects of 6‐Gin on contractile and relaxation function

**Figure 8:**
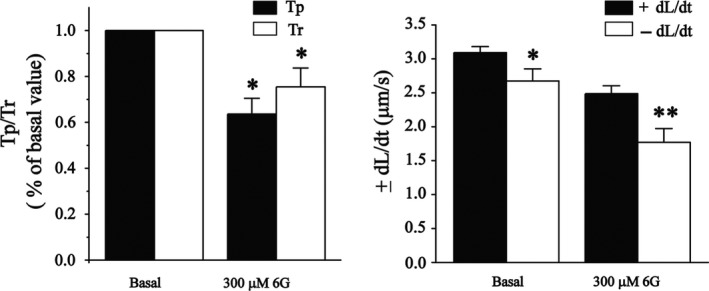
Effects of 6‐Gin on contractile and relaxation function. Data of Tp, Tr, and the maximum velocity of contraction‐relaxation in the absence and presence of 300 μmol/L 6‐Gin. Data are expressed as means ± *SEM* (*n* = 8 cells). *p < 0.05, **p < 0.01, versus control

The time to 50% of the peak (Tp) describes the speed of myocyte shortening or Ca^2+^ elevation, the time to 50% of the baseline (Tr) is a parameter of cellular relaxation or Ca^2+^ reuptake. 6‐Gin at 300 μmol/L decreased the Tp and Tr (*p < *0.05) (Figure 8). Also, 6‐Gin at 300 μmol/L decreased the maximum velocity of contraction‐relaxation (±d*L*/d*t*) (*p < *0.05 or *p < *0.01) (Figure 8).

## 4 DISCUSSION

Ginger is a food and traditional medicine used for centuries. 6‐Gin is a major active ingredient in ginger and possesses a variety of interesting pharmacological effects. However, the molecular mechanisms of 6‐Gin on cardio‐protection have yet been reported to the best of our knowledge. This work reports intracellular I_Ca‐L_, contractility, and Ca^2+^ transients in isolated rat ventricular myocytes to detail the molecular mechanisms of 6‐Gin underlying its cardio‐protective effects.

The isolated myocyte model provides a specific opportunity to observe physiological adaptations of cardiac function. Calcium is a ubiquitous signal that is responsible for a broad range of cell activities (Berridge, Bootman, & Roderick, 2003; Clapham, 2007). Ca^2+^ is rapidly removed from the cytoplasm via pumps (Pozzan, Rizzuto, Volpe, & Meldolesi, 1994) and exchangers (Blaustein & Lederer, 1999), for example, the Ca^2+^‐ATPase pumps and Na^+^/Ca^2+ ^exchangers. This is then reported via signals. Internal calcium stores are held in the ER or SR membrane systems of muscle cells (Berridge, Lipp, & Bootman, 2000). Calcium ion release is then controlled by various channels including the inositol‐1, 4, 5‐trisphosphate receptor (InsP3R) and ryanodine receptor (RYR) families (Berridge, 1993; Clapham, 1995). Ca^2+^ passing through the calcium channel is important for cardiac electrical activity and the excitation–contraction coupling of cardiac muscle. The principal activator of these channels is Ca^2+ ^itself.

There is a depolarizing current when calcium ions flow into cells and calcium current flow after the calcium channels open. Other mechanisms for influx of Ca^2+^, for example, Na^+^/Ca^2+^ exchange, can also lead to depolarization and increase cytosolic calcium. A trace of calcium entry from the calcium channel causes more release of Ca^2+^ from the SR, that is, Ca^2+^‐induced Ca^2+^ release (CICR). The CIRC hypothesis (Fabiato, 1983) states that the release of calcium from the SR is not only promoted by a rapid elevation of the Ca^2+^ activity (d[Ca^2+^]j/d*t*) but also inactivated by a moderate or prolonged elevation of [Ca^2+^]_i_. Myocardial contractility was trigged mainly by cytosolic calcium ions entry through calcium channels (Blaustein & Lederer, 1999), which can mediate excitation–contraction coupling. Cardiac muscle is activated by the depolarization‐dependent Ca^2+^ current and the release of calcium from SR that elevates myoplasmic calcium and allows the myofilaments to contract (Atwater, Rojas, & Vergara, 1974).

Alternatively, Ca^2+^ stores can help generate Ca^2+^ transients. This sequence of biochemical events is triggered by a Ca^2+^ transients, beginning with Ca^2+^ binding to troponin C. Cell shortening resulting from a rise in [Ca^2+^]_i_ is also activated following repolarization from positive potentials. Measurements of cell shortening, especially of [Ca^2+^]_i_, show that the activation process closely mirrors both the time course and the voltage dependence of the Ca^2+^ current. The Ca^2+^ current in cardiac cells does not act primarily as a direct activator of the contractile filaments but that it acts indirectly by releasing Ca^2+^ from the SR. A wave of depolarization opens the T‐type channels first followed by LTCCs. Calcium antagonists (CCAs) act by changing the mode of channel opening from long‐duration to shorts. Thus, CCAs lower the rate at which Ca^2+^ enters via the LTCCs. VER is a CCA and interfered with the calcium‐dependent processes.

Our data suggest that 6‐Gin reduces the I_Ca‐L_ (Figure 3) in a concentration‐dependent manner with an IC_50_ of 31.25 μmol/L in cardiomyocytes. Figure 4 shows that the I‐V relationship or the reversal potential of I_Ca‐L _did not change. Furthermore, the contractility and Ca^2+^ transients were inhibited by 6‐Gin (Figures 6 and 7). Also, 6‐Gin at 300 μmol/L reduced the I_Ca‐L _in ischemic ventricular myocytes (Figure 3d–f). Ischemia causes membrane depolarization, calcium influx in ischemic cells is increased. Elevated intracellular calcium accelerates the activity of several ATP‐consuming enzymes, which further depletes already marginal cellular energy stores, making the heart even more susceptible to ischemic damage (Undrovinas & Maltsev, 1998). Our data suggest that 6‐Gin could inhibit the increase in [Ca^2+^]_i _via decreasing the extracellular Ca^2+^ influx. Excitation–contraction coupling in all cardiac cells requires Ca^2+^ influx, therefore the inhibitory effects of 6‐Gin on contractility may through the reduction on Ca^2+^ influx. Collectively, these results detail the cardio‐protective effects of 6‐Gin on rat ventricular myocytes as well as and its cellular mechanism.

## 5 CONCLUSIONS

These results clearly indicate that 6‐Gin inhibits the Ca^2+^ transients and contractility of cardiomyocytes. This is mainly via inhibition of the L‐type Ca^2+^. This restricts Ca^2+^ flow into the ventricle myocytes and decreases [Ca^2+^]_i_. The findings of the present study provide new perspectives for further research on pharmacology of 6‐Gin as a possible candidate for the treatment of cardiovascular diseases.

## CONFLICT OF INTEREST

The authors declare no conflict of interest.

## ETHICAL STATEMENT

All animal care and experimental protocols were ethically reviewed and approved by the Ethics Committee of Hebei University of Chinese Medicine.
